# Minimizing outbreak through targeted blocking for disease control: a community-based approach using super-spreader node identification

**DOI:** 10.1038/s41598-023-41460-3

**Published:** 2023-08-30

**Authors:** Amir Sheikhahmadi, Mehri Bahrami, Hero Saremi

**Affiliations:** 1 Department of Computer Engineering, Islamic Azad University, Sanandaj Branch, Sanandaj, Iran; 2 Department of Mathematics, Islamic Azad University, Sanandaj Branch, Sanandaj, Iran

**Keywords:** Immunology, Health care, Engineering

## Abstract

The COVID-19 pandemic has caused significant disruptions to the daily lives of individuals worldwide, with many losing their lives to the virus. Vaccination has been identified as a crucial strategy to combat the spread of a disease, but with a limited supply of vaccines, targeted blocking is becoming increasingly necessary. One such approach is to block a select group of individuals in the community to control the spread of the disease in its early stages. Therefore, in this paper, a method is proposed for solving this problem, based on the similarity between this issue and the problem of identifying super-spreader nodes. The proposed method attempts to select the minimum set of network nodes that, when removed, no large component remains in the network. To this end, the network is partitioned into various communities, and a method for limiting the spread of the disease to communities is proposed by blocking connecting nodes. Four real networks and four synthetics networks created using the LFR algorithm were used to evaluate the control of the disease by the selected set of nodes using each method, and the results obtained indicate better performance of the proposed method compared to other methods.

## Introduction

Controlling the spread of infectious diseases has emerged as a critical global health challenge in the 21st century, with significant threats posed by major epidemics such as COVID-19, SARS, dengue fever, MERS, and Ebola^1,2^. Prompt and effective interventions are crucial for minimizing the impact on public health during outbreaks. One vital step in managing these diseases is contact tracing, which helps identify potentially infected individuals since diseases can spread through person-to-person contact^3^. Technological advancements in information and communication have opened doors to more efficient contact tracing methods, such as the development of radio frequency identification (RFID) systems^4^ that enable real-time recording of contact activities.

Targeted immunization (TI)^5^ is a commonly employed method to control epidemic outbreaks. It focuses on identifying and immunizing individuals or groups at high risk of transmitting the disease to a larger population. Effective disease outbreak modeling, coupled with appropriate TI response measures, plays a crucial role in containing the spread of infectious diseases^6,7^. Allocating optimal resources and developing effective immunization strategies become particularly important when resources are limited, and vaccines are scarce. The protection of healthcare workers (HCWs), who are at the forefront of medical care during outbreaks, becomes paramount, especially in healthcare facilities where diseases like SARS and MERS can rapidly propagate through person-to-person contact.

In this research, our specific focus is on the spread of infectious diseases through person-to-person contact in healthcare facilities, with an emphasis on protecting HCWs. We approach this problem by formulating it as an equivalent problem to influence maximization (IM) in social network analysis^8^. The IM problem involves targeting a specific number of influential individuals, referred to as “seed” nodes, to maximize the spread of influence in social networks^9^. On the other hand, the TI problem aims to identify a set of individuals to minimize the effects of epidemic spread. By considering the reward of protecting the population, TI can be transformed into a maximum reward problem, which is equivalent to the IM problem.

In the propagation of phenomena within complex network structures, two critical issues arise: (1) the optimal selection of a set of network nodes to minimize the spread rate (referred to as “super-blockers”)^2,10^) the selection of a set of nodes to maximize the propagation rate (known as “Top-spreaders”)^11,12^. While some recent research treats these two issues as equivalent, closer examination reveals that super-blockers and Top-spreaders differ in nature. super-blockers are nodes that, when removed from the network, minimize the size of connected components, whereas Top-spreaders are nodes that, when selected as initiators, maximize the average emission rate of propagation.

In this study, our proposed method focuses on the selection of super-blockers, addressing the first problem mentioned above. super-blockers are network nodes whose removal minimizes the spread of diseases by reducing the size of connected components. We recognize that super-blockers and Top-spreaders represent distinct groups of nodes in the network. Therefore, our method aims to select the minimum set of nodes for removal from the network, ensuring that no extensive components remain.

The contributions of this paper are as follows:

- Introducing a novel greedy method for selecting the minimum number of nodes to effectively separate different communities, preventing the spread of disease or contamination between communities.
- Presenting a method to accurately measure the spreading power of nodes within each community, taking into account weighted connections and a novel approach based on a combination of *k*-shell centrality and the sum of weights of neighboring and neighboring-of-neighbor nodes. This method enhances the identification of influential spreaders.
- Proposing a modified version of the SIR (Susceptible-Infectious-Recovered) model to assess the impact of vaccination on controlling the spread of disease through vaccinated super blocker nodes. this modified SIR model enhances the understanding and evaluation of the impact vaccination strategies on disease spread within healthcare networks.

The remainder of this paper is organized as follows: In "Related work" section, a comprehensive review of related literature is provided. In “Proposed method” section presents the methodology employed in this research, including the formulation of the problem as an influence maximization problem and the selection of super-blockers. In "Evaluation" section, the experimental setup, evaluation of the proposed method, and presentation of results and discussions are presented. Finally, "Conclusion" section concludes the paper by summarizing the contributions, highlighting the significance of the research, and suggesting future directions for further investigation in this field.

## Related work

Several studies have investigated the impact of network structure and community dynamics on disease spread and control in complex networks. In their work on epidemic spread on patch networks with community structure, researchers^13^ investigate the influence of community structure on the spread of diseases within human metapopulation networks. They highlight the significant impact of community structures on disease reproduction rates, emphasizing the importance of mitigation strategies such as movement restrictions and vaccinations.

The identification of influential spreaders in complex networks for disease spread and control is investigated by authors^14^. They examine four metrics (betweenness, degree, H-index, and coreness) to measure node centrality and construct disease spreading models. The study reveals the varying effectiveness of these metrics in different network types, providing insights into node importance for disease transmission and control. Addressing the challenge of early pandemic mitigation, researchers introduce the dynamic Community-based Mitigation strategy (ComMit)^15^. ComMit offers a blind community-based approach that reduces infection peaks by 73% and shortens infection duration by 90%, even in scenarios with steady-state infections. In optimizing vaccine resources for disease control, authors propose the Community Priority based vaccine distribution Strategy (CPS)^16^. CPS assigns priority to communities based on centrality measures, effectively controlling disease spread by immunizing critical groups within communities.

Node removal vulnerability of networks is examined by authors^17^, who propose a greedy algorithm for minimizing the size of the largest component. The study demonstrates the effectiveness of their approach in reducing the size of the largest component with a relatively small number of node removals. The resilience of the dengue virus network is investigated by researchers^18^. They analyze the network’s robustness by strategically removing links using different centrality measures. The study highlights the dependence of node and link robustness on the network’s topology and provides insights into epidemic control.

A review on link and node removal in real social networks^19^ explores the impact of link and node removal on network responses. The review emphasizes the practical relevance of studying social network dynamics, particularly during the COVID-19 pandemic, and suggests the need to consider link weights for accurate analyses. Researchers introduce the Modular Centrality framework^20^ to identify influential spreaders in networks with non-overlapping communities. They extend this framework to networks with overlapping communities, proposing the Overlapping Modular Centrality as a superior measure for node centrality in such networks. The study on immunization of networks with non-overlapping community structure^21^ focuses on deterministic strategies for controlling epidemics. The proposed strategies consider node connectivity, link types, and community sizes, demonstrating superiority over alternatives that do not consider community structure.

A systematic review on community detection algorithms in healthcare applications^22^ highlights the increasing popularity of social network analysis techniques and community detection algorithms in health informatics. Authors^23^ propose a hybrid method for identifying multi-spreader users in social networks for viral marketing, using the k-shell measure and selecting a group of superior nodes to maximize influence spread.

In the field of complex networks, a method based on link prediction is introduced^24^ for identifying influential spreader nodes. The method incorporates diffusion power calculations and demonstrates superior performance in enhancing the spread of influence. Additionally, the paper on super-spreaders and super-blockers based community evolution tracking in dynamic social networks^10^ presents a two-stage approach for detecting and monitoring communities. The proposed method accurately detects dynamic network communities and identifies critical evolutionary events based on core node characteristics.

The *k*-shell method^25^ measures the importance of nodes in a graph based on their proximity to the core. It employs the *k*-shell decomposition method, which iteratively determines the centrality of nodes. Starting with a counter *k* set to 1, nodes with degree 1 are moved to the 1-shell of the graph until there are no more such nodes. These moved nodes are assigned $ks = 1$. The process is repeated with *k* set to 2, moving nodes with degree less than or equal to 2 to the 2-shell, and so on, until all nodes have been moved and their centrality determined. Nodes with higher *k*-shell numbers are considered more important, as they are closer to the core of graph.

Another study^26^ focuses on identifying super-spreaders in epidemics and information transmission within multiplex networks. The proposed coupling-sensitive centrality measure accounts for structural and dynamic couplings between communication and physical contact layers, demonstrating superior accuracy compared to traditional centrality measures.

In^27^, the influence of population support on preventive measures during epidemic outbreaks is explored. The researchers utilize the susceptible-infected-recovered (SIR) model to study adaptive behaviors influenced by individuals’ perception of the epidemic environment. Their findings indicate that local awareness can increase the epidemic threshold, delay the occurrence of peak prevalence, and decrease the size of outbreaks. However, the effectiveness of local awareness is reduced in networks with high heterogeneity, as highly connected individuals are less responsive to the epidemic environment. Strategies focusing on socially active individuals can enhance outbreak mitigation.

In the Distance-based coloring (DCD) method^28^, the graph is initially colored. To achieve this, nodes are sorted in descending order based on their degrees. The node with the highest degree is assigned a color. Subsequently, the next node is checked, and if it is a neighbor of the previously colored node, it is placed in a different color group. This process continues until all nodes are assigned a color. The control set is then formed from nodes with the maximum degree that are not neighbors, meaning they have different colors compared to the previously colored nodes. These nodes are identified as members of the control set.

In the Heuristic clustering (HC) method^29^ nodes are clustered. For this purpose, first, the centers of the clusters are randomly selected. Then other nodes are assigned to the appropriate cluster. In the next steps, each cluster is sorted and the new center of the cluster is selected. This process continues until no changes occur in the clusters. Cluster centers are selected as members of the outbreak control group.

In the Community based K-Shell (CKS)^30^, a method is introduced to select the initial seed set from super-spreaders, with the goal of reducing overlap among highly connected nodes in terms of their *k*-shell centrality. The approach utilizes the *k*-shell decomposition method to organize nodes within each community based on the number of shells. The proposed CKS+ method evaluates the spread power of nodes within each community and chooses influential nodes with the highest ranks for the propagation process.

Community Finding Influential Node (CFIN) algorithm^31^ focuses on identifying k users from a network’s community structure to maximize influence spread. CFIN consists of two parts: seed selection and local community spreading. Seed nodes are selected from detected communities using a community detection algorithm, and independent influence spread occurs within each community starting from the final seed nodes. In Table 1 the summary of related work presented.

**Table 1:** Summarization of related work.

Authors	Category	Method	Application	Evaluation metric
^13^	Epidemiology	Mathematical analysis	Impact of community structure on epidemic spread	Reproduction rate
^14^	Epidemiology	Centrality metrics	Node importance in disease transmission and control	epidemic threshold
^15^	Epidemiology	Community-based strategy	Early pandemic mitigation	Infection peak reduction, infection duration
^16^	Epidemiology	Community priority strategy	Vaccine distribution for disease control	Disease spread control, severed connections
^17^	Network vulnerability	Greedy algorithm	Minimizing the size of the largest network component	Size of the largest component
^18^	Epidemiology	Network robustness analysis	Resilience of dengue virus network	Node and link robustness
^19^	Social network dynamics	Review	Impact of link and node removal on network responses	Largest connected component, network efficiency
^20^	Complex networks	Modular centrality	Identifying influential spreaders in networks with community structure	Node centrality
^21^	Epidemiology	Immunization strategies	Controlling epidemics in networks with non-overlapping communities	Epidemic control effectiveness
^22^	Healthcare informatics	Systematic review	Application of community detection algorithms in healthcare	N/A (Review)
^23^	Social network analysis	Hybrid method	Identifying influential nodes for viral marketing	Spread of influence
^24^	Complex networks	Link prediction	Identifying influential spreader nodes for information spreading	Spread of influence
^10^	Dynamic social networks	Two-stage approach	Detecting and monitoring communities in dynamic networks	Accuracy of community detection, identification of evolutionary events
^26^	Multiplex networks	Coupling-sensitive centrality	Identifying super-spreaders in epidemics and information transmission	Accuracy of super-spreader identification
^27^	Adaptive Prevention	Susceptible-Infected-Recovered (SIR) Model, Local and Global Awareness Rules	Heterogeneous Networks	Epidemic Threshold, Prevalence Peak Delay, Outbreak Size Reduction
^30^	Complex networks	Initial Seed Selection (CKS+)	Identifying influential spreader nodes for information spreading	Spread of influence
^31^	Complex networks	Community Finding Influential Node	Identifying influential spreader nodes for information spreading	Spread of influence

## Proposed method

**Figure 1:**
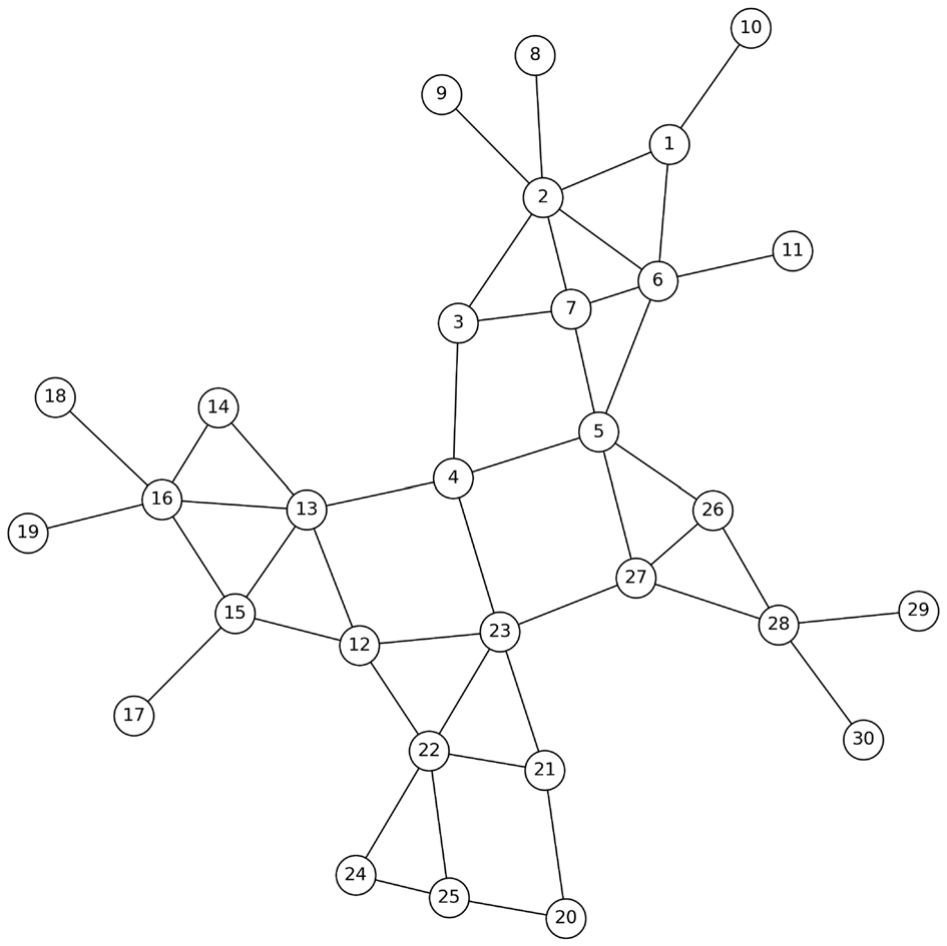
A small example of a graph modeled society.

One method that proves useful in problem-solving across various fields involves creating a small sample that represents the problem at hand. This approach is equally applicable to the challenge of identifying individuals within a network who can control the spread of an entire community when blocked. In order to illustrate this method, we will utilize a small network depicted in Fig. 1.

As illustrated in Fig.  1, the network can be divided into distinct communities based on their characteristics. By doing so, we can then focus on seeking the desired nodes within each community. These nodes possess the capability to effectively control the spread of entire community when they are blocked. In the subsequent sections, we will delve into the comprehensive examination of the general steps involved in this proposed method, along with the individual components comprising each step.

**Figure 2:**
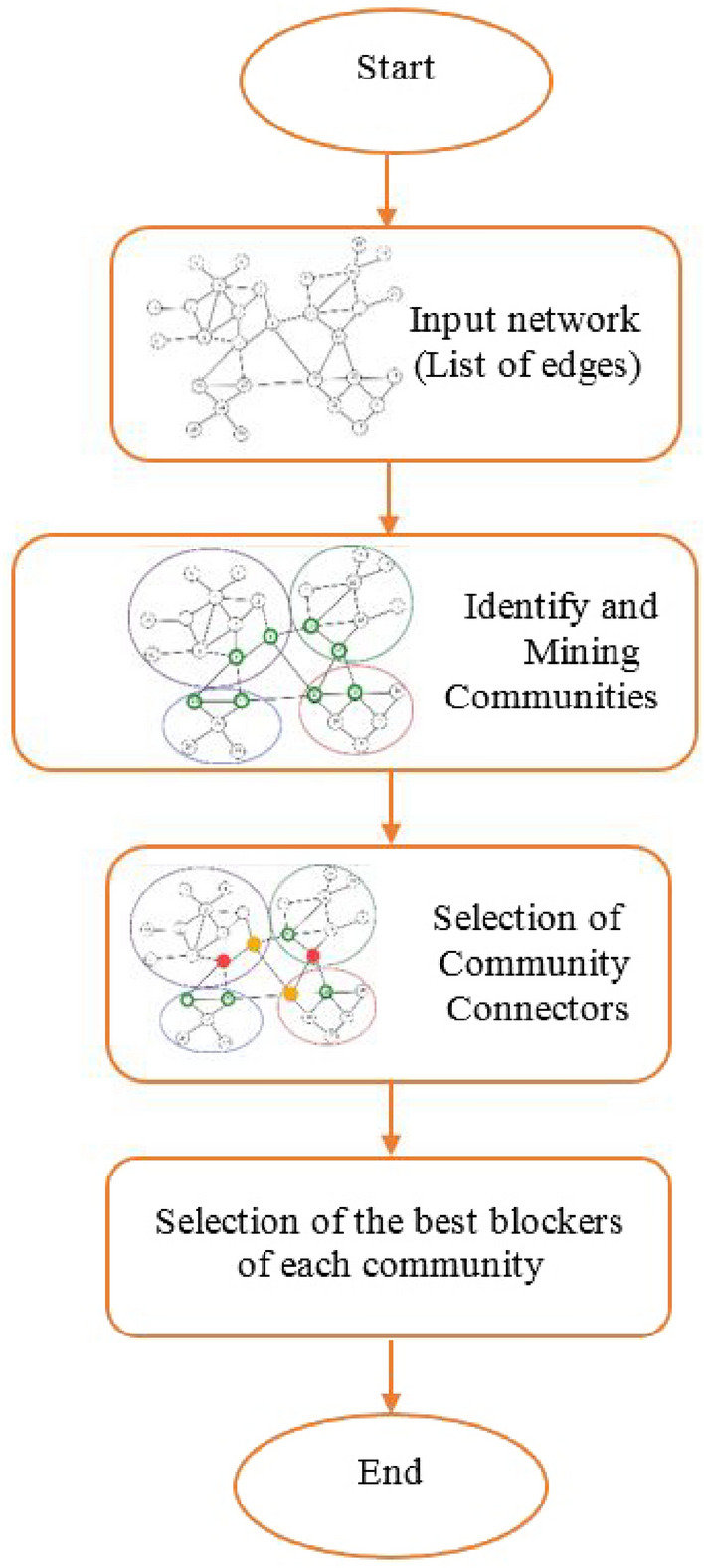
Procedure of the proposed method.

Our proposed method for selecting super-blockers to Control Infectious Diffusion (SBCID) is depicted in Fig. 2. In the following sections, we will delve into the general steps of the proposed method, providing a comprehensive examination of each component.

### Input network

As shown in Fig. 2, the first step in the proposed method is to read the input network. Considering that in most existing networks, the information is in the form of a list of available edges. Therefore, the data of input network (Fig. 1), is in the Table 2.

**Table 2:** Part of the input data set of the proposed method.

From , To	
1 , 2	
1 , 6	
1 ,10	
2 ,3	
2, 6	
2,7	
2 , 8	
2, 9	
3,7	
3,4	

According to Table 2, generally, the data sets used are in csv format (data separated by commas), in which the first number indicates the source node number and the second number indicates the destination node number.

### Extracting network communities

Complex networks, including social networks, possess unique characteristics that differentiate them from other networks. One important characteristic is the tendency of nodes to establish stronger connections with specific nodes in the network. These connections form what we refer to as “communities,” which are regions of the network that exhibit a higher density of edges compared to other areas. Identifying and utilizing these communities can significantly enhance the effectiveness of our proposed method. Given that many diseases spread through interpersonal contact, and considering the increased communication and interaction within society, these communities become particularly relevant in understanding and controlling epidemics.

To accomplish this, in paper, we utilize a method introduced in^32^ for extracting communities from the input graph. The method consists of two phases:

1. Initially, nodes are individually assigned to communities. Nodes are then repositioned to neighboring communities if it enhances modularity. This process is repeated until no further improvements are achievable.
2. In the second phase, a new network is constructed using the communities from the first phase. The links between the new nodes are weighted based on the links between nodes in the corresponding communities. This generates a hierarchical structure of communities, progressively reducing the meta-communities with each iteration until the maximum modularity is attained.

**Figure 3:**
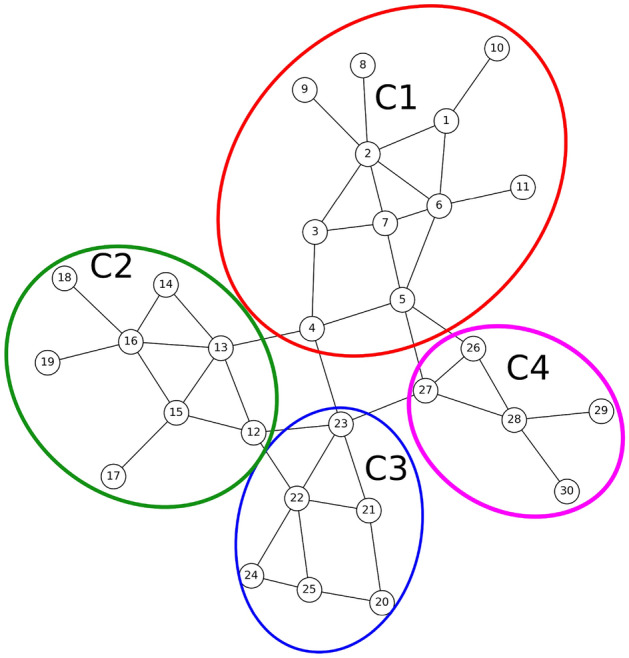
The result of running the community detection algorithm on the sample graph of Fig. 1.

The results of applying the community detection algorithm to our sample graph are depicted in Fig. 3. The method successfully identifies four distinct communities:

- Community C1: Consisting of nodes 1 to 11;
- Community C2: Consisting of nodes 12 to 19;
- Community C3: Consisting of nodes 20 to 25;
- Community C4: Consisting of nodes 26 to 30.

### Selection of community connectors

**Figure 4:**
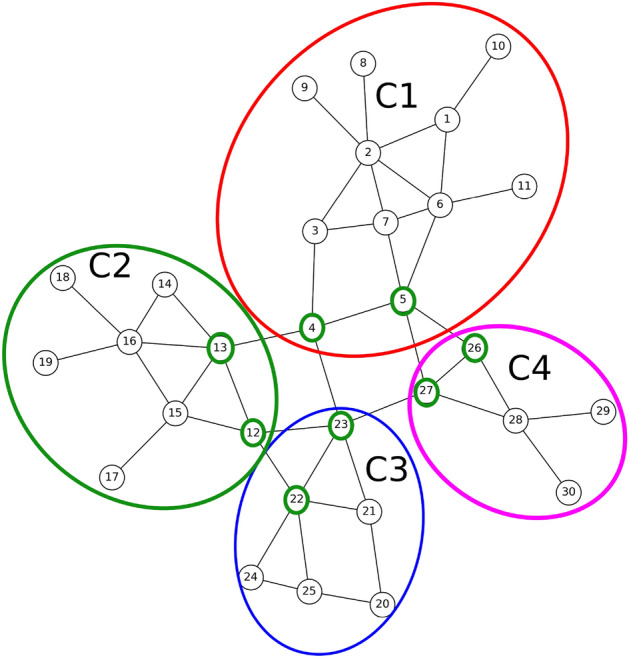
Nodes connecting different societies.

**Figure 5:**
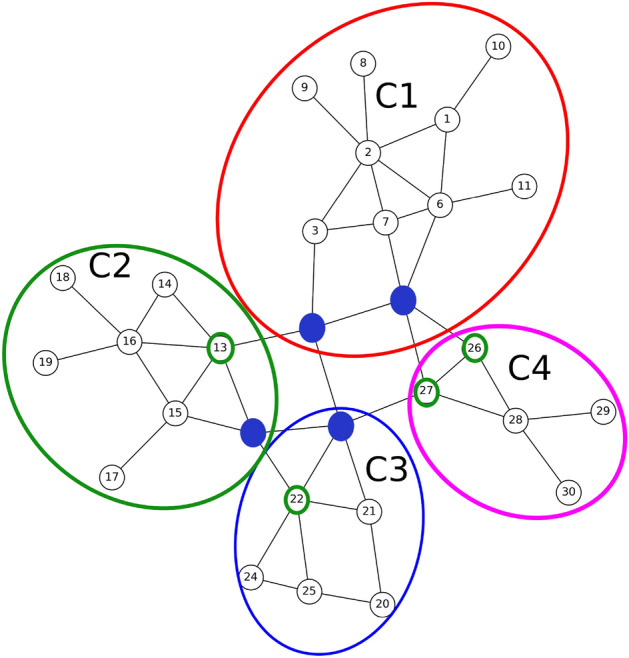
Nodes that by removing them, each society is separated from other societies.

By examining the communities depicted in Fig. 3, it becomes apparent that certain nodes within these communities could potentially belong to different communities. For instance, node 5, initially placed in community C1, has connections with nodes 26 and 27 in community C4. Similarly, node 4 has connections with node 13 in community C2 and node 23 in community C3. The presence of these nodes and their diverse connections can facilitate the rapid spread of diseases throughout the network. Therefore, in this phase, these nodes are identified. Each node’s set of neighbors is locally checked, and if a neighbor from another community is found within this set, the node is identified as a connector. The nodes identified as connectors are visually differentiated by a distinct color, as shown in Fig. 4. The selection steps of these nodes are as follows:
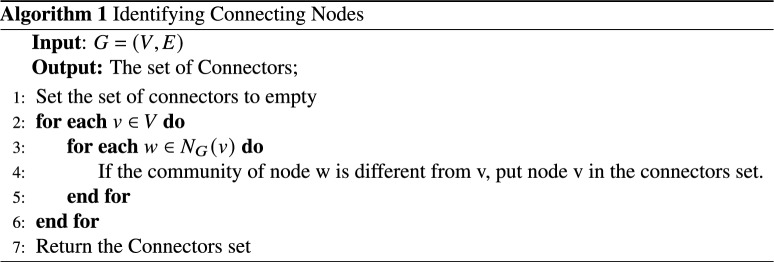

In the sample network, nodes 4, 5, 12, 13, 22, 23, 26 and 27 play the role of connecting communities C1 to C4. The existence of these nodes can cause the spread of disease from one community to another. Therefore, in order to isolate the communities, in the first step, it is necessary to completely cut off the communication between them. For this purpose, these connecting nodes are specified as the initial set and an effort is made to determine the minimum node that, by removing them, the communities will be completely separated. By considering Fig. 5, it can be seen that by removing node 5, the connection between C1 community and C4 is disconnect. By removing node 4, the connection between C1 community and C2 and C3 communities will be cut off. By removing node 12, the connection between C2 community and C3 is cut off, and finally, by removing node 23, the connection between C3 and C4 communities is cut off. The result is shown in Fig. 5.

The steps for arbitrary networks are given in Algorithm 2. In this algorithm, which receives connecting nodes as input; First, it sorts these nodes in descending order of the number of their friends in different communities. For example, node 4 of community C1 has two friends in communities C2 and C3, which will be removed from the set of connectors of node 13 by selecting and removing it. Because it no longer connects societies. By repeating this procedure, the isolating nodes are specified one after the other and the connectors set is empty. In this way, the minimum number of nodes that can completely separate each society from other societies are determined. Obviously, if the number of members of a society is very small, they can be omitted or they can be made a part of a larger society.
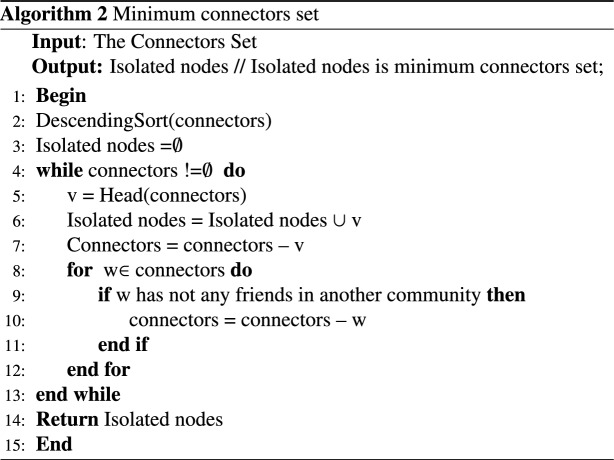


### Determining the top spreaders in each community to be blocked

After separating each society and removing the nodes that are communication between the nodes of one society and other societies. It is necessary to specify the nodes that should be selected for blocking in each community. Considering that a lot of contact and communication can be the main cause of the spread of respiratory diseases. Firstly, the communication edges between the nodes of the society, which represent the contact and mutual relations between the nodes, were weighted. For this purpose, due to the lack of real communication between network nodes, the concept of number of common neighbors and also the degree of two-headed nodes are used to measure the communication weight. In the other words, the probability of communication between two nodes that have more common neighbors increases. Also, the edge between two nodes that connects more neighbors is more important. Eq. (1) is used to calculate the number of common neighbors of edge nodes,

1 $$\begin{aligned} CN(u,w)=|N(u)\cap N(w)|, \end{aligned}$$

where *N*(*u*) and *N*(*w*) represents the set of neighbors of nodes *v* and *w* and Eq. (2) is used to weight the communication edges between the nodes of each community.

2 $$\begin{aligned} W_{v,w}=(CN(u,w)+1)(deg(v)+deg(w)) \end{aligned}$$

**Figure 6:**
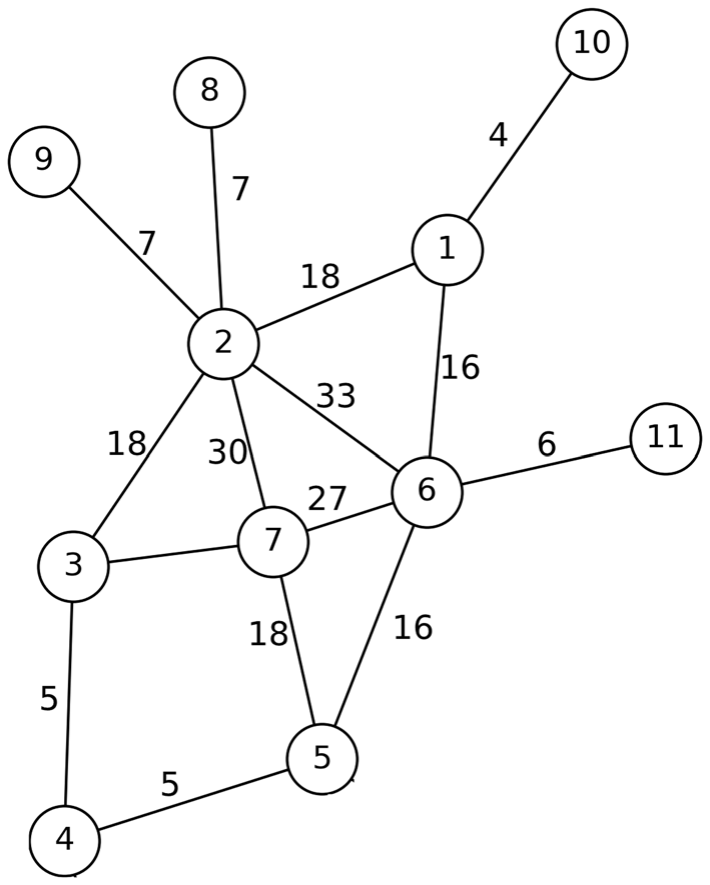
Weighted edges of community C1.

In Eq. (2), the value of the common neighbor is added to one so that if the two end vertices of the edge do not have a common neighbor, the weight of the edge is not zero. Fig. 6 shows the result of weighting edges between nodes of community *C*1.

After weighting the edges between the nodes of each community, it is necessary to calculate the outbreak power of the nodes of each community and select the nodes with higher power to be blockaded. For this purpose, a semi-local method is presented, in which not only the weight of the edges of each node with its neighbors is considered, but also the weight of the edges between the neighbors and the neighboring neighbors is considered. Therefore, Eq. (3) has been used to compute the outbreak of each node *u*:

3 $$\begin{aligned} SP(u)=ks(u)+\sum _{\begin{array}{c} v\in N(u)\\ v \ is \ not\ blocked \end{array}}\sum _{\begin{array}{c} s\in N(v)\\ \ s\ is \ not \ blocked \end{array}}W_{v,s} \end{aligned}$$

In Eq. (3), *ks* is the *k*-shell number in which the node is located, and the weight of the edges of the node with neighbors of level 1 and 2 is also considered. It should be mentioned, to reduce the overlap of edge weight towards the previously blocked nodes, we do not consider it.

**Figure 7:**
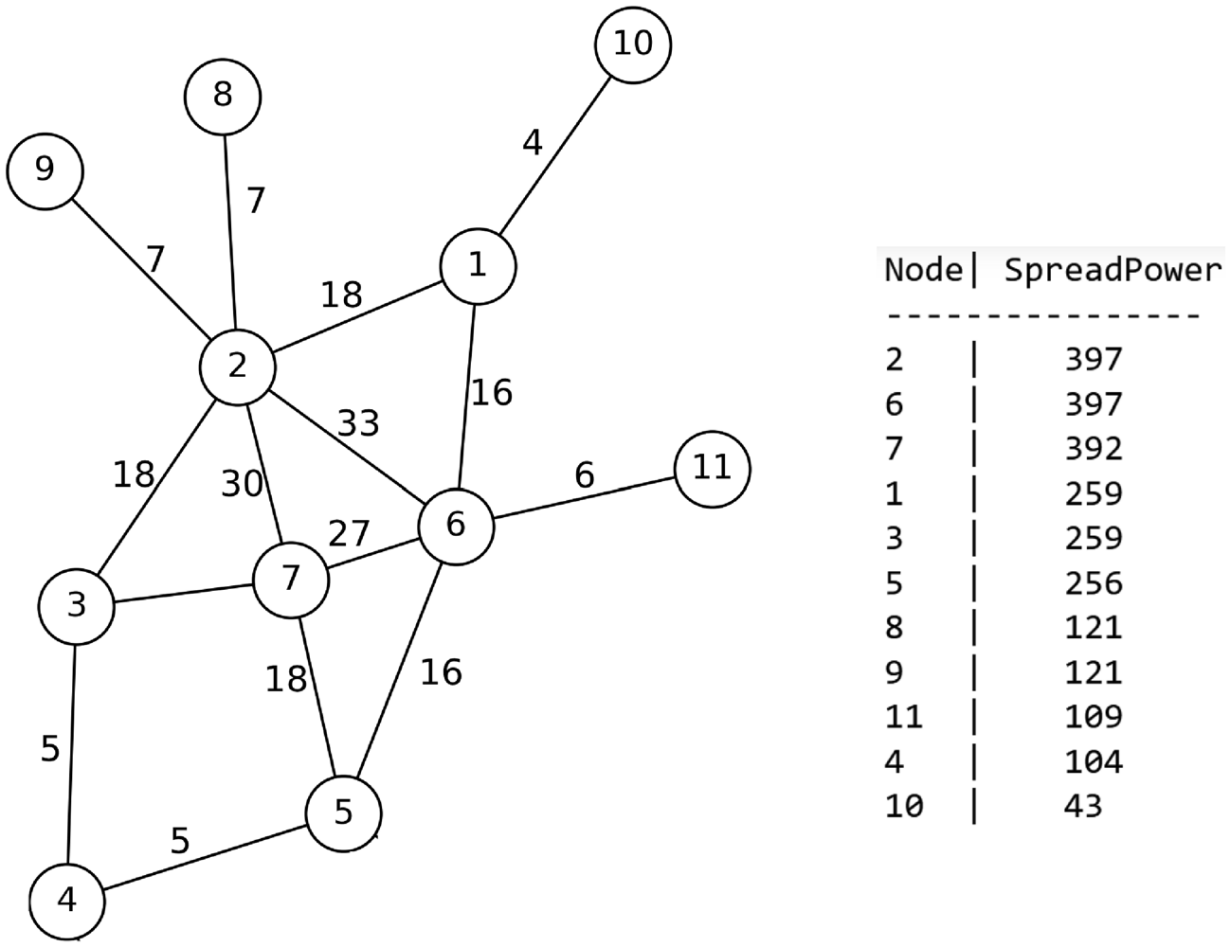
Community C1 and the outbreak rate of each of its nodes.

In community *C*1 shown in Fig. 7, nodes 8, 9, 10 and 11 are located in *k*-shell 1 and nodes 1 to 7 are located in *k*-shell number 2. That is, $ks_1 = \{8, 9, 10, 11\}$ and $ks_2 = \{1, 2, 3, 4, 5, 6, 7\}$. The results obtained from the outbreak of the nodes of this community are shown in descending order in Fig. 7.

The obtained results show that nodes 2, 6 and 7 are nodes that can infect more nodes in case of contamination or disease. Therefore, depending on the amount of vaccine available, these nodes can be selected as the main candidates for blocking. This procedure has been implemented in other identified communities and in each community, a number of nodes with the highest outbreak are selected for primary blocking. In this way, the network can be protected with a much smaller number of doses and the outbreak can be controlled in the very initial steps. The general procedure of the proposed method is given in Algorithm 3.
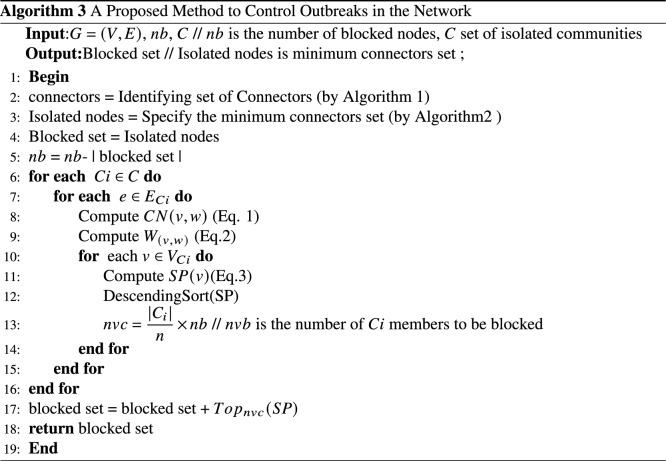


### Time complexity of the proposed method

To evaluate the time complexity of the proposed algorithm, we analyze each step:

1. The community detection Algorithm 1 used in the method has a time complexity of *O*(*nlogn*) when applied to a graph with n nodes.
2. Identifying set of Connectors (Algorithm 1 ): The time complexity is approximately $O(|V| * d)$, where |*V*| is the number of nodes in the graph G, and d is the average node degree.
3. Specify the minimum connectors set (Algorithm 2 ): The time complexity depends on the number of connectors, assumed to be *c*.
4. DescendingSort(SP): Sorting the SP list in descending order takes $O(c * log(c))$ time.
5. Calculations within loops involving *CN*(*v*, *w*) and *W*(*v*, *w*) take constant time, *O*(1).
6. Nested loops iterating through communities and edges take linear time in the worst case.
7. *SP*(*v*) calculation involves nested summations, resulting in $O(d^2)$ time in the worst case.
8. Subsequent sorting of SP takes $O(d^2 * log(d^2))$ time.
9. Calculating *nvb* involves division and multiplication, taking constant time, *O*(1).
10. Adding the top *nvc* elements from the *SP* list to the blocked-set takes *O*(*nvc*) time.

Overall, the time complexity of the algorithm is given by:

$$T(n) = O(nlogn) + O(|V| * d) + O(c * log(c)) + O(d^2 * log(d^2)) + O(nvc).$$

Since the community detection algorithm (*O*(*nlogn*)) dominates the time complexity, we can approximate it as: $T(n) \approx O(nlogn)$. Therefore, the overall time complexity of the algorithm is approximately *O*(*nlogn*), where *n* is the number of nodes in the graph.

## Evaluation

In this section, the effectiveness of the proposed method, Super-blockers to Control Infectious Diffusion (SBCID), is evaluated through a comparative analysis with several established methods for selecting influential nodes to enhance influence spread in the network. The networks used in the experiments are introduced, and a detailed explanation of the parameters employed in generating artificial networks is provided, considering their relevance to the datasets. The evaluation criteria are then outlined, and the outcomes derived from comparing the methods are presented. Lastly, the conclusion is presented at the end of the section.

To gauge the effectiveness of our proposed approach, SBCID, we subjected it to comparison with a variety of methods, including K-Shell (KS)^25^, Distance-based coloring (DCD)^28^, Mixed Core, Semi-local Degree, and Weighted Entropy (MCDE)^23^. Furthermore, we incorporated several community detection-based methods, such as Heuristic clustering (HC)^29^, Community based K-Shell (CKS)^30^, and the Community Finding Influential Node (CFIN) algorithm^31^. All methods underwent implementation using Python and were executed on a computer equipped with a Core i7 2.6 GHz CPU and 32 GB RAM.

### Datasets

To evaluate the proposed method, four real-world datasets were used: Zachary Karate Club^33^, High School Friendship^34^, Jazz Musicians^35^, and Adolescent Health networks^36^. In the Zachary Karate Club dataset, nodes represent club members, and edges represent relationships between members. The High School Friendship dataset consists of male students and their friendship connections. In the Jazz Musicians dataset, each node represents a musician, and edges indicate band collaborations. The Adolescent Health dataset represents students and their friendship connections. Real network data were collected from the Koblenz University Konect Project^34^.

To complement the real datasets, synthetic networks were generated using the Lancichinetti-Fortunato-Radicchi (LFR) technique^37^. The LFR benchmark algorithm produces artificial networks with predefined communities to compare community detection methods. It considers diversity in node degrees and community sizes using power laws ($\gamma$ and $\beta$). The mixing parameter $\mu$ controls the proportion of edges between different communities, reflecting network noise. At $\mu =0$, all links stay within the same community, creating isolated clusters. At $\mu =1$, all links connect nodes from different communities, resulting in a structure-less network. The adaptability of the LFR benchmark in adjusting $\mu$ is valuable for evaluating community detection methods.

To investigate the impact of community structure on methods, two versions of the LFR-200 datasets were used with parameters $\gamma =3$, average degree $({\bar{k}})=5$, $|V|=200$, and mixing parameters $\mu =0.2$ and $\mu =0.7.$ Similarly, for the LFR-1000 dataset, the parameters were $\mu =2$, average degree $({\bar{k}})=12, |V|=1000$, and mixing parameters $\mu =0.2$ and $\mu =0.7$. Further details about the datasets, including the number of nodes |*V*|, number of edges |*E*|, average degree ${\bar{k}}$, and maximum degree *Max*(*k*), can be found in Table 3.

**Table 3:** Detailed information of datasets.

Dataset	|*V*|	|*E*|	$({\bar{k}})$	*Max*(*k*)	Assortatiivity
Zachary Karate Club	34	78	4.588	17	-0.4756
High School	70	366	10.457	23	0.0829
Jazz Musicians	198	2742	27.697	100	0.0202
LFR-200 $(\mu =0.2)$	200	423	5	26	0.2188
LFR-200 $(\mu =0.7)$	200	423	5	28	0.2209
LFR-1000 $(\mu =0.2)$	1000	6529	12	122	-0.0743
LFR-1000$(\mu =0.7)$	1000	6523	12	132	0.0795
Adolescent health	39	12.969	10.215	36	0.2512

### Evaluation criteria

In the context of identifying influential nodes to maximize diffusion in networks, various well-known models are used to calculate the extent of diffusion. Notable among these models are the Linear Threshold (LT) model, the Independent Cascade (IC) model, and the Susceptible-Infected-Recovered (SIR) diffusion model^38^. The SIR model is widely employed in the literature to simulate spreading processes and determine the spreading ability of each node as an indicator of its importance in the network. In the SIR model, a group of individuals in the network are initially infected with a disease and can then infect their neighboring nodes with a probability of $\beta$. Subsequently, these nodes themselves recover with a probability of $\gamma$ or leave the infected group due to other factors like death.

In the proposed method, the goal is to measure the impact of node selection in controlling the spread of disease. Therefore, some modifications are made to the basic SIR model. A fourth set, called the blocked set *V*, is introduced in addition to the susceptible set *S*, infected set *I*, and recovered set *R*. Nodes selected by the methods are considered members of the blocked set, and it is assumed that they will not be included in the infected collection due to blocking and will not contribute to the further spread of the disease. Algorithm 4 presents the modified SIR diffusion model used to measure the effect of node selection in controlling the spread of disease. Algorithm 4, the modified SIR diffusion model is given to measure the effect of the selection of nodes in controlling the spread of the disease.
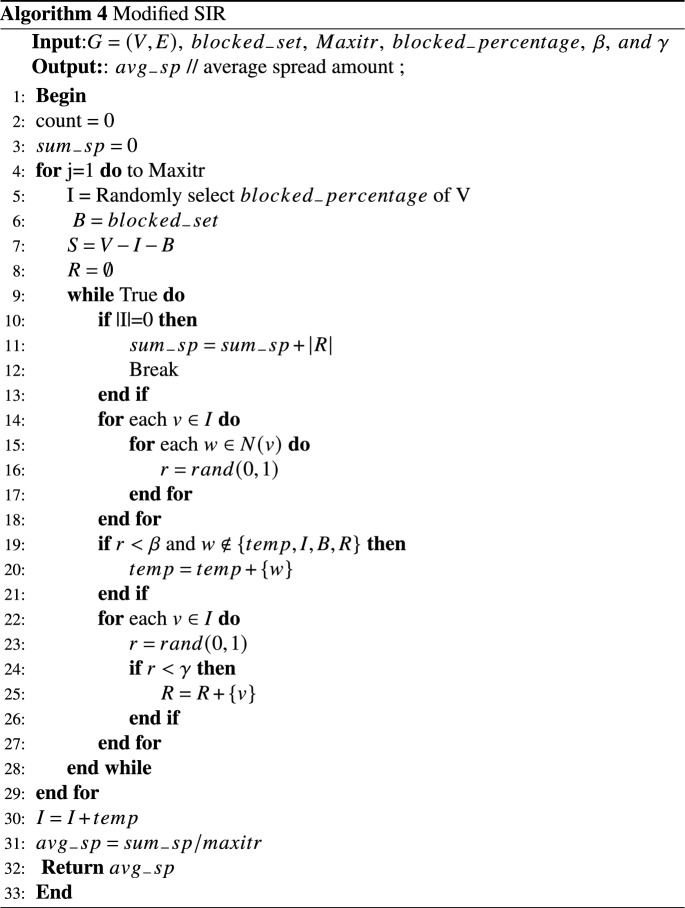


### The results obtained from the control of the outbreak

In this section, we compare the impact of the set of nodes selected by each method for blocking. To conduct this comparison, we first run each method on the datasets from Table 3 and determine a set of nodes for initial blocking. Next, we examine the effect of choosing these nodes in controlling the spread of disease in networks. To achieve this, as explained in the previous section, we utilize the modified SIR model to measure the outbreak of the disease. Since the model involves random elements, in order to reflect real-world scenarios, we run it 1000 times and report the average results.

**Figure 8:**
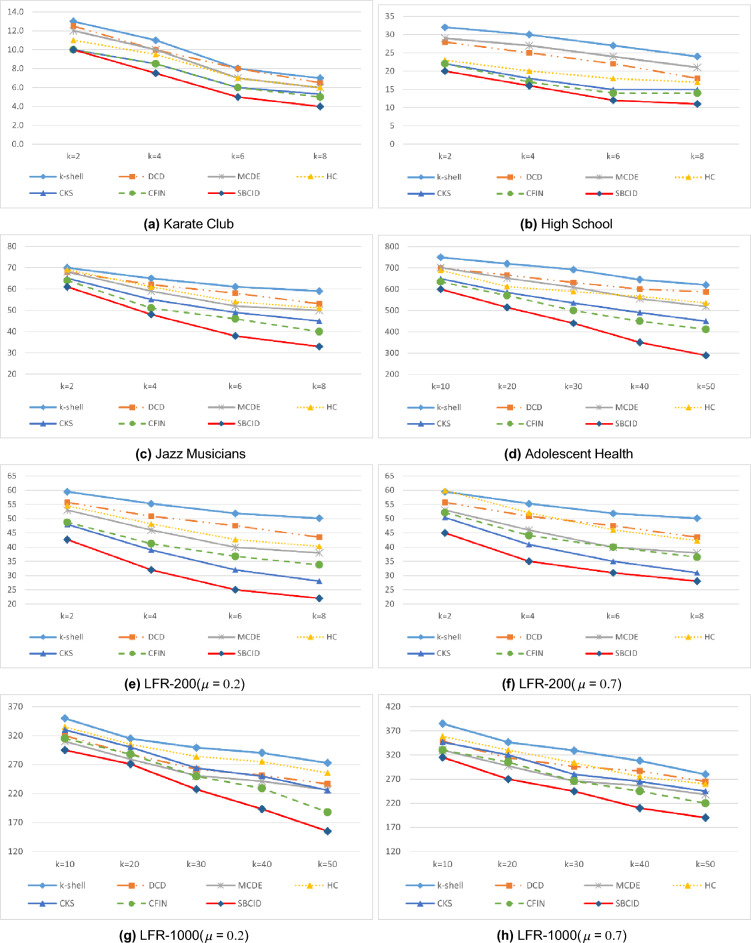
Disease outbreak control by blocking a part of the nodes in different datasets $\beta =0.1$, $\gamma =1$.

In the first experiment, the results of which are depicted in Fig. 8a–h, we set the number of initially infected nodes to $10\%$ of the total population, with $\beta = 0.1$ and $\gamma = 1$ as the specified parameters. In Fig. 8, the horizontal axis represents the number of vaccinated nodes, while the vertical axis represents the number of recovered individuals at the end of modified SIR model.

The results presented in Fig. 8 demonstrate that the SBCID method proposed in this paper outperforms other methods in controlling the outbreak in all the datasets. This superiority can be attributed to the SBCID method’s ability to promptly isolate communities from each other, effectively limiting the spread of contamination. Moreover, the method efficiently identifies and rapidly vaccinates powerful blocking nodes within each community, further contributing to its effectiveness in disease control.

It is important to highlight that the LFR-200 and LFR-1000 datasets with $\mu =0.7$ (Fig. 8f and h) exhibit a less well-separated community structure, with numerous inter-community edges connecting nodes from different communities. As a consequence, both the proposed method and other community-based methods show slightly weaker performance in comparison to other datasets. Nevertheless, the proposed method in this paper has managed to maintain its superiority in these datasets, displaying resilience to these challenges and demonstrating its effectiveness in controlling the spread of disease.

In the subsequent evaluation of the methods, we consider $15\%$ of the network nodes to be initially infected (I) and $\gamma = 0.5.$ The SIR model requires a minimum value of $\beta$ known as the threshold ${\beta _{th}}$^39^. This is calculated as $\frac{{\bar{k}}}{\bar{K^2}}$, where ${\bar{k}}$ and $\bar{k^2 }$ denote the mean first and second-order degrees of the nodes in graph, respectively. As suggested by Bae and Kim in^39^, the SIR diffusion model requires the spreading probability value $\beta$ to exceed the threshold $\beta _{th}$. The corresponding $\beta$ values utilized in this experiment are detailed in Table 4 for each of the datasets.

**Table 4:** Corresponding $\beta$ values used for each datasets.

Dataset	$\beta _{th}$	$\beta$
Zachary Karate Club	0.129	0.15
High School	0.026	0.05
Jazz Musicians	0.0953	0.12
LFR-200$(\mu =0.2)$	0.18	0.19
LFR-200 $(\mu =0.7)$	0.17	0.18
LFR-1000 $(\mu =0.2)$	0.053	0.06
LFR-1000 $(\mu =0.7)$	0.050	0.06

**Figure 9:**
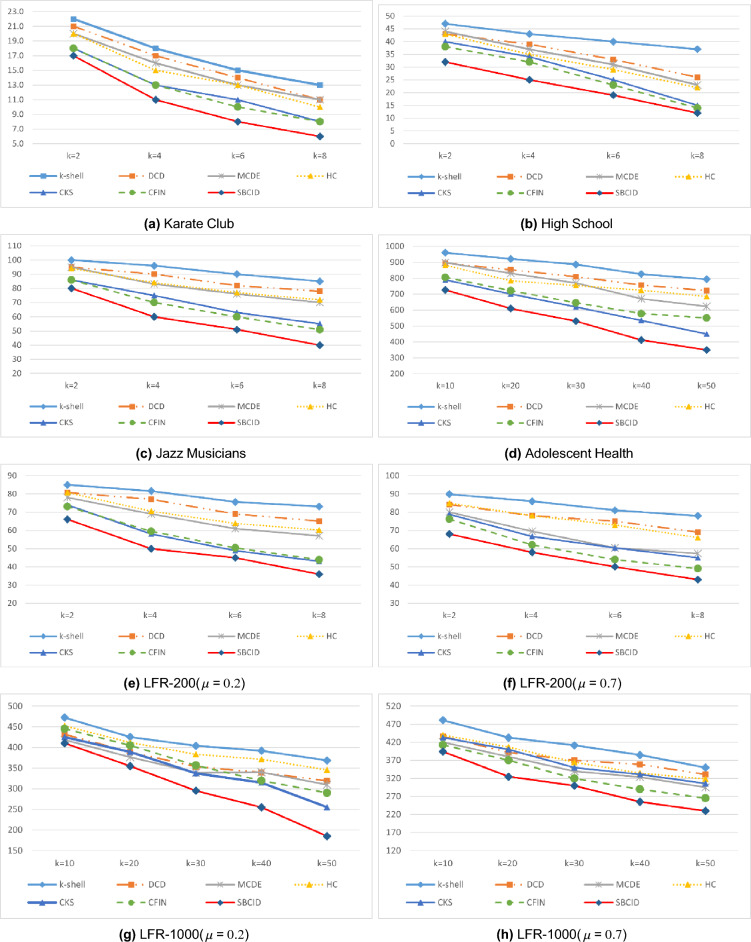
Disease outbreak control by blocking a part of the nodes in different datasets $\beta > {\beta }_{th}$, $\gamma =0.5$.

The results obtained, as shown in Fig. 9 , unequivocally confirm the superior performance of the proposed SBCID method across all datasets. This notable improvement can be attributed to the precise identification of nodes that require early blocking, particularly due to their higher number of connections with other nodes in the graph. Furthermore, the SBCID method efficiently facilitates the swift isolation of communities from each other, thereby significantly enhancing its effectiveness in controlling the spread of disease.

## Conclusion

This paper introduces a method for selecting individuals to block, whose vaccination can effectively control the spread of disease in the network during the initial stages. The proposed approach aims to identify the minimum set of nodes that, when removed, will not leave any large components in the network. It involves separating each community and removing nodes acting as inter-community connectors, followed by considering weighted edges between nodes within each community. Subsequently, the spreading power of nodes within each community is calculated, and nodes with higher spreading power are selected for vaccination. Evaluations on real and artificial datasets demonstrate that the proposed method outperforms other approaches in disease control. This superiority is attributed to its ability to rapidly isolate communities and limit contamination to distinct groups, as well as quickly vaccinate influential blocking nodes within each community. For future work, adjusting simulation model parameters based on different conditions and diseases can enhance the method’s usability as a recommended. Additionally, exploring and improving each component of the proposed method, such as community detection, connector identification, and measuring node spreading power within communities, can further refine its performance.

## Data Availability

The datasets analyzed during the current study are available in the Konect repository, Konect.cc.
